# Effectiveness of the Korean National Cancer Screening Program in reducing breast cancer mortality

**DOI:** 10.1038/s41523-021-00295-9

**Published:** 2021-06-28

**Authors:** Eunji Choi, Jae Kwan Jun, Mina Suh, Kyu-Won Jung, Boyoung Park, Kyeongmin Lee, So-Youn Jung, Eun Sook Lee, Kui Son Choi

**Affiliations:** 1 Graduate School of Cancer Science and Policy, National Cancer Center, Goyang, Republic of Korea; 2 National Cancer Control Institute, National Cancer Center, Goyang, Republic of Korea; 3 Department of Medicine, Hanyang University College of Medicine, Seoul, Republic of Korea; 4 Center for Breast Cancer, National Cancer Center, Goyang, Republic of Korea

**Keywords:** Cancer screening, Breast cancer

## Abstract

High incidences of breast cancer (BC) are reported in Asian women in their forties, and it is not clear whether mammographic screening reduces mortality among them. This study evaluated the effect of BC screening on mortality in Korea. We conducted a nationwide prospective cohort study of women invited to the Korean National Cancer Screening Program (KNCSP) between 2002 and 2003 (*N* = 8,300,682), with data linkage to the Korea Central Cancer Registry and death certificates through 2014 and 2015, respectively. Exposure to mammographic screening was defined using a modified never/ever approach. The primary study outcome was adjusted mortality rate ratio (MRR) for BC among screened and non-screened women estimated by Poisson regression. An adjusted MRR for all cause-death other than BC was examined to account for selection bias in the cohort. BC incidence rates for screened and non-screened women were 84.41 and 82.88 per 100,000 women-years, respectively. BC mortality rates for screened and non-screened women were 5.81 and 13.43 per 100,000 women-years, respectively, with an adjusted MRR for BC of 0.43 (95% CI, 0.41−0.44). The adjusted MRR for all-cause death excluding BC was 0.52 (95% CI, 0.52−0.52). The greatest reduction in BC mortality was noted for women aged 45−54 years, and there was no observable reduction in mortality after the age of 70 years. In conclusion, the KNCSP has been effective in reducing BC mortality among Korean women aged 40−69 years.

## Introduction

Breast cancer in women is a major public health problem, eliciting the highest incidences of cancer (age-standardized rate [ASR], 46.3) and cancer death (ASR, 13.0)^1^, as well as the highest cancer disability-adjusted life-years (14.9 million)^2^. Geographic and temporal trends in the incidence and mortality of breast cancer, however, differ greatly across age groups, countries, ethnicities, etc. These variations are, in part, attributable to genetics, risk factors, or access to and the effectiveness of screening programs and treatment. With the observed efficacy of mammographic screening in reducing breast cancer mortality in Western countries, the Korean National Cancer Screening Program (KNCSP) for breast cancer was launched in 2002 to provide biennial mammography for women aged 40 years and older^3^. Although breast cancer screening services have been offered free of charge or at a co-payment of 10% of the total cost of the procedure depending on a participant’s income status^4^, screening uptake rates still remain lower than recommended levels (9.4% in 2002 and 59.7% in 2015)^5,6^.

Breast cancer incidence and mortality rates for Korean women are distinctly different from those for Western women. According to Korea’s latest cancer statistics, breast cancer was the most common cancer (ASR, 55.6), other than thyroid cancer, and the fifth leading cause of cancer death (ASR, 5.5); moreover, both the incidence and mortality of breast cancer have gradually increased without reductions over the past few decades^7^. Different from Western women, age-specific incidence rates in Korean women peak around the ages of 45−54 years and then decrease with age^8^. These observations of higher breast cancer incidence among younger women are specific to Asian countries, where breast densities tend to be high. Higher breast density makes it difficult to detect tumors in breast tissue via mammography^9^ and has been shown to be associated with an increased risk of breast cancer^10^. Indeed, Park et al. reported that the risk of breast cancer increased as much as 9.4 times among Korean women who were younger and had extremely dense breasts^11^. Moreover, mammographic screening sensitivity and discriminatory performance are significantly lower in Korean women with higher breast density, regardless of modality^12^; significantly higher cancer detection rates were also reported among women with higher breast density than those with lower breast density^13^.

Despite noted differences in the tissue characteristics and risk for breast cancer among Asian women, only a few studies have reported on the effectiveness of organized mammographic screening programs. Greatly limiting their generalizability, these studies only describe several intermediate outcomes^14–16^ over a short period^17^ at regional levels^14^. Moreover, the benefits of breast cancer screening in reducing breast cancer mortality, the results of which have primarily been derived from studies in Western countries, vary from none to 60%, depending on the study design and the length of follow-up^18^.

The primary aim of the study was to examine reductions in breast cancer mortality for screened versus non-screened women invited to undergo biennial mammographic screening under the KNCSP. We followed up the entire Korean women aged 40 years and older who were invited to the KNCSP for breast cancer between 2002 and 2003 and followed through 2015 (14-year follow-up). To account for potential selection bias, we applied a modified ever/never approach to define the exposure status to screening (i.e., non-screened cohort versus screened cohort) using the concept of a changeable group and further performed mathematical adjustment using breast cancer-specific and all-cause except breast cancer mortality rates. Additionally, we assessed the effect of participating in the program on breast cancer incidence over the follow-up period. The evidence obtained from the cohort design ought to reflect the “real-world” effectiveness of the program in terms of relative risk.

## Results

### Participant characteristics

From 2002 to 2014, 6,125,603 (45.96%) women underwent breast cancer screening at least once (Table 1). The mean ages at entry to the cohort were 54.08 and 56.87 years for non-screened and screened women, respectively. The age at entry to the cohort for non-screened women was measured at the year of their first screening invitation, and that for screened women was at the time of their first screening attendance. Overall, a large proportion of women in the screened cohort (41.7%) underwent their first breast cancer screening between the age of 45 and 54 years. Over 70% of the screened women attended screening more than once during follow-up (Supplementary Table 1). As a modified never/ever approach, we applied the concept of a changeable group for women who can change their screening exposure status (see “Methods”). 76% of the women in the changeable group underwent their first screening between their second and fourth screening invitations from 2004 to 2009 (Supplementary Table 2).

**Table 1:** Baseline characteristics of the screened and non-screened cohorts in the KNCSP for breast cancer, Republic of Korea.

	Total		Non-screened cohort		Screened cohort		*P* value
	*N*	(%)	*N*	(%)	*N*	(%)	
Total population	13,326,868	(100.00)	7,201,265	(100.00)	6,125,603	(100.00)	
Mean age at cohort enrollment (years; SD)	55.17 (10.39)		54.08 (10.96)		56.87(9.44)		<.0001
5-year age groups at cohort enrollment							
40−44	2,488,854	(18.68)	1,949,151	(27.07)	539,703	(8.81)	<.0001
45−49	2,215,733	(16.63)	1,129,829	(15.69)	1,085,904	(17.73)	
50−54	2,558,101	(19.20)	1,088,358	(15.11)	1,469,743	(23.99)	
55−59	1,460,066	(10.96)	626,267	(8.70)	833,799	(13.61)	
60−64	1,800,592	(13.51)	933,902	(12.97)	866,690	(14.15)	
65−69	1,153,157	(8.65)	568,263	(7.89)	584,894	(9.55)	
70−74	1,118,722	(8.39)	608,112	(8.44)	510,610	(8.34)	
75−79	531,643	(3.99)	297,383	(4.13)	234,260	(3.82)	
National Health Insurance							
Upper 50%	8,242,352	(61.85)	4,547,529	(63.15)	3,694,823	(60.32)	<.0001
Lower 50%	4,444,694	(33.35)	2,309,418	(32.07)	2,135,276	(34.86)	
Medical Aid Program	639,822	(4.80)	344,318	(4.78)	295,504	(4.82)	

*SD* standard deviation.

### Breast cancer incidence in the screened and non-screened cohorts

**Fig. 1:**
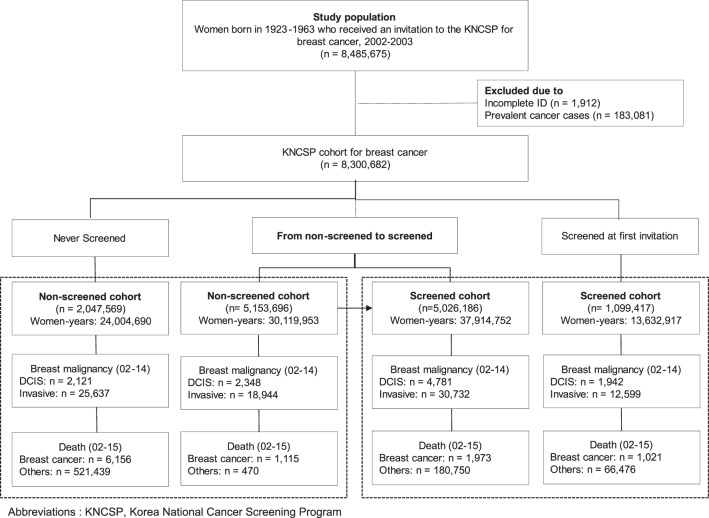
Flow diagram for the study-cohort in the Korean National Cancer Screening Program. Exposure to breast cancer screening was defined using a modified never/ever approach by which women were considered ever screened after theirfirst screening attendance (see "Methods").

The average times to follow-up in the screened and non-screened cohorts were 8.42 (median: 8.66) years and 7.52 (median: 6.63) years, respectively (Fig. 1). The time from initial screening attendance to breast cancer detection was 3.81 years on average among screened women (interquartile range: 0.97−6.04). During the follow-up period, 43,331 and 44,581 invasive breast cancers were diagnosed in the screened and non-screened cohorts, respectively; 6,723 and 4,469 in situ breast cancers were found in the screened and non-screened cohorts, respectively (Table 2 and Supplementary Table 3). The proportion of localized cancer was significantly higher among screened women (64.7% and 59.0%, respectively for overall and invasive breast cancer only) than that among non-screened women (54.5% and 48.3%) (Supplementary Table 3). Crude invasive breast cancer incidence rates were 84.41 and 82.88 per 100,000 women-years in the screened and non-screened cohorts, respectively, which resulted in an adjusted incidence rate ratio (IRR) of 1.09 (95% CI, 1.08−1.11). The IRR of total breast cancer (in situ and invasive) was 1.15 (95% CI, 1.14−1.17). The cumulative incidence of in situ and invasive breast cancer among screened women was much higher than that in non-screened women in the earlier period; however, the difference in the cumulative incidences of breast cancer in situ and invasive breast cancer between the screened and non-screened cohort began to decrease after 11 and 12 years of follow-up, respectively (Supplementary Table 4).

**Table 2:** Crude incidence rates and adjusted incidence rate ratios for breast cancer and crude mortality rates and adjusted mortality rate ratios for breast cancer and all causes of mortality except from breast cancer between the screened and non-screened women in the KNCSP.

	No. of cases	Total women-year	Crude rates^a^	Adjusted rate ratios^b^
*Incidence*				
Invasive breast cancer				
Screened cohort	43,331	51,331,087	84.41	1.09 (1.08−1.11)
Non-screened cohort	44,581	53,789,002	82.88	
Both ductal carcinoma in situ and invasive breast cancer^c^				
Screened cohort	50,054	51,296,678	97.58	1.15 (1.14−1.17)
Non-screened cohort	49,050	53,753,069	91.25	
*Mortality*				
Breast cancer				
Screened cohort	2,994	51,547,670	5.81	0.43 (0.41−0.44)
Non-screened cohort	7,271	54,124,644	13.43	
All cause of mortality except from breast cancer				
Screened cohort	247,226	51,547,670	479.61	0.52 (0.52−0.52)
Non-screened cohort	521,909	54,124,644	964.27	

^a^Per 100,000 women-years.

^b^Adjusted for age at enrollment of the cohort (continuous) and for National Health Insurance status (categorical).

^c^Lobular carcinoma included.

### Effect of the KNCSP in breast cancer mortality reduction

**Fig. 2:**
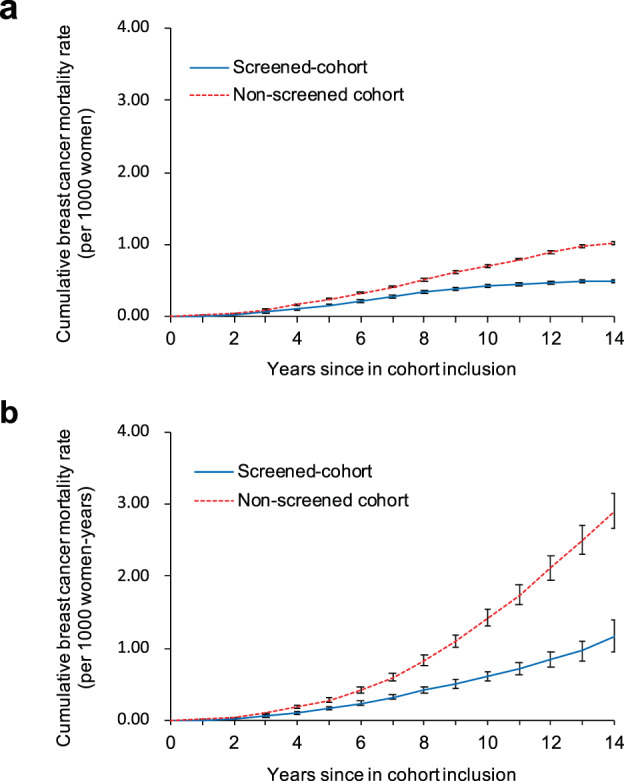
Cumulative mortality rates between the screened and non-screened cohorts in the KNCSP, 2002−2015. **a** Cumulative mortality rates with 95% confidence intervals. **b** Nelson−Aalen estimates of the cumulative mortality rates with 95% confidence intervals.

Crude breast cancer mortality rates were 5.81 and 13.43 per 100,000 women-years in the screened and non-screened cohorts, respectively (Table 2). The adjusted mortality rate ratio (MRR) of breast cancer was 0.43 (95% CI, 0.41−0.44). When taking into account for MRR of all-cause deaths except from breast cancer (MRR; 0.52, 95% CI, 0.52−0.52), the net benefit (see statistical analysis in “Methods”) of breast cancer mortality reduction among women aged 40−79 years from the KNCSP was 18%. The difference in crude cumulative breast cancer mortality rates between the screened and non-screened women tended to increase beginning at 3 years after inclusion in the cohort (Fig. 2 and Supplementary Table 5).

The reduction in breast cancer-specific mortality among women aged 40−69 years was significant, with an adjusted MRR of 0.41 (95% CI, 0.39−0.43). The net benefit of breast cancer mortality reduction was 22.08% after mathematical adjustment for self-selection bias (Table 3). The net benefit of reduced breast cancer mortality was largest in women aged 45−49 years (31.98%), followed by those aged 50−54, 55−59, 60−64, and 65−69 years. However, there was no significant mortality reduction among women aged 70−79 years after adjusting for self-selection bias. The number needed to screen (NNS) to prevent one death from breast cancer mortality was 1,920 (Supplementary Table 6). NNS was smallest among women aged 50−54 years at 1,623, followed by women aged 55−59 years and 45−49 years, at 1,688 and 1,691, respectively, reflecting the large magnitude of the beneficial effect of the KNCSP. Women aged 40−44 years showed the highest NNS value at 3,633, which we attributed to low mortality in both non-screened and screened women, indicating a substantial economic burden on the KNCSP with screening for breast cancer in this group.

**Table 3:** Reductions in breast cancer mortality according to adjusted mortality rate ratios (MRRs) for breast cancer and all causes of death except breast cancer between the screened and non-screened women in the KNCSP.

	Breast cancer				All cause of death except from breast cancer				Mortality reduction of breast cancer^a^
	No. death in screened cohort	No. death in non-screened cohort	CRR	MRR (95% CI)	No. death in screened cohort	No. death in non-screened cohort	CRR	MRR (95% CI)	
Total									
40−69	2,621	6,381	0.42	0.41 (0.39−0.43)	132,120	214,191	0.63	0.52 (0.52−0.52)	22.08
40−79	2,994	7,271	0.43	0.43 (0.41−0.44)	247,226	521,909	0.50	0.52 (0.52−0.52)	17.97
5-year age groups at cohort enrollment									
40−44	377	1,898	0.50	0.47 (0.42−0.52)	5,517	19,892	0.70	0.60 (0.58−0.62)	22.42
45−49	567	1,258	0.38	0.38 (0.34−0.42)	11,062	16,074	0.58	0.56 (0.54−0.57)	31.98
50−54	658	1,158	0.38	0.37 (0.34−0.41)	18,644	22,656	0.55	0.53 (0.52−0.54)	29.90
55−59	378	655	0.38	0.37 (0.33−0.42)	17,499	21,357	0.54	0.52 (0.51−0.53)	28.58
60−64	393	851	0.43	0.41 (0.37−0.46)	35,912	59,562	0.56	0.53 (0.52−0.54)	22.22
65−69	248	561	0.41	0.40 (0.35−0.47)	43,486	74,650	0.54	0.52 (0.52−0.53)	23.41
70−74	246	570	0.56	0.55 (0.47−0.64)	65,750	162,518	0.52	0.52 (0.51−0.52)	−6.07
75−79	127	320	0.61	0.60 (0.49−0.74)	49,356	145,200	0.52	0.52 (0.52−0.53)	−14.54
National Health Insurance									
Upper 50%	1,566	4,114	0.44	0.44 (0.41−0.46)	112,067	275,933	0.47	0.51 (0.50−0.51)	13.61
Lower 50%	1,150	2,590	0.41	0.42 (0.39−0.45)	93,127	170,933	0.51	0.51 (0.51−0.52)	18.90
Medical Aid Program	257	567	0.40	0.40 (0.35−0.47)	42,032	74,983	0.46	0.55 (0.55−0.56)	27.26

Adjusted for age at enrollment of the cohort (continuous) and for National Health Insurance status (categorical).

*CRR* crude rate ratio, *MRR* mortality rate ratio.

^a^Mortality reduction = [(MRR for all cause of death except from breast cancer) – (MRR for breast cancer)] ÷ (MRR for all cause of death except from breast cancer) × 100.

## Discussion

Over 14 years, the KNCSP has afforded a significant reduction in breast cancer mortality among women aged 40−69 years, with a net benefit of 22% after adjusting for selection bias. The greatest net benefit of 30% was observed among Korean women aged 45−59 years who bear the highest incidence of breast cancer. Relatively smaller net benefit (22%) was detected among women in 60−69 years, which was partly driven by a lower incidence of breast cancer and a smaller screening impact among the age groups. The NNS to prevent one death from breast cancer ranged from 1,623 to 3,633. Our results present the effectiveness of organized breast cancer screening in breast cancer mortality reduction, based on the nationwide cohort with the longest follow-up in Asian countries.

In North America and Western Europe, mammographic screening for women in their forties is controversial. Still, results from the Malmo trial and Gothenburg trial showed 37% and 40% reductions in breast cancer mortality with two-view mammographic screening every 18−24 months, respectively^19,20^. The UK Age trial also demonstrated a 50% reduction among the intervention group with annual mammography within 10 years from breast cancer diagnosis^21^. Our results estimated 53% and 62% reductions in breast cancer mortality among women aged 40−44 and 45−49 years, respectively, and the relatively higher magnitude of reduction may stem from discrepancies between the trials and our observational design. Depending on the screening intervals and screening methods (e.g., either one- or two-view mammographic screening), the effect sizes varied from none to 60% for women in their forties however^18^, observational studies in Sweden and Canada showed similar risk reductions as ours^22,23^.

For women aged 50 years and older, the adjusted MRR for breast cancer among screened to non-screened women was 0.38−0.41, values which were similar to those in other studies comparing breast cancer mortality between screened versus non-screened women^22,24–27^, but higher than studies comparing mortality between invited and uninvited women^28–32^. A recent Taiwanese cohort study compared breast cancer mortality among women who participated or did not participate in screening, matched by propensity score for baseline risk factors, and reported an estimated 41% reduction among women aged 50−69 years. However, their short follow-up period of 5 years and the lack of women aged under 50 years prevent direct comparisons with our results^17^. Our estimates of mortality reduction for women aged 50 years and older were comparable with previous studies from Norway, Canada, and Italy^22,24,33^.

The harms of screening in terms of overdiagnosis need to be considered. To quantify overdiagnosis, adjustment for the underlying incidence of breast cancer in the absence of screening and lead time is essential. Though the scale of overdiagnosis was not discernible in our study, there is the possibility of overdiagnosis, because the proportions of breast cancer in situ were constantly higher among screened women than non-screened women. However, a recent paper reported that the annual percent increase of invasive breast cancer was lowered in the most recent 5 years, and this observation may be, in part, attributable to the effect of the KNCSP^34^. In addition, cases diagnosed ahead by the lead-time, were intuitively observable by examining the decreased gaps between the cumulative incidences of breast cancer in screened and non-screened women after 11 years in our data. The decreased gap in breast cancer incidence after 11 years from screening implementation might be due to “a compensatory drop” after lead-time among screened women, as well as “the actual screening effect” by removing in situ breast cancer. Given that a cancer diagnosis is brought forward through screening, the incidence among screened women declines after screening has stopped as a compensatory drop. In addition, the removal of in situ breast cancer through the mammography test under the KNCSP might reduce the development of subsequent invasive breast cancers. Accurate correction of lead-time will enable examinations of net overdiagnosis conferred by screening in Korea.

In statistical analysis, we used a changeable group to assign a screened or non-screened cohort of women according to their first exposure to screening^24^. Because the screening rates were low early after the implementation of the KNCSP, our prospectively defined exposure status reduced the magnitude of selection bias, compared with the method of determining screening status at initial screening invitation^35^. Two exceptions were applied among women diagnosed with breast cancer before the first screening attendance and the prevalent cases at the introduction of the screening program. Their transfer to a non-screened cohort was logical to estimate screening effects and did not much inflate mortality rates from a non-screened cohort due to small number of death cases among them. Using the concept of a changeable group also ensured our conservative estimation of the observed screening effect through the KNCSP, by augmenting women-year (denominator) into the non-screened cohort. Still, further efforts should be made to improve screening rates and thus increase the impact of screening. Previous papers have reported various facilitators of mammographic screening attendance in Korea, including psychological and socio-demographic factors, such as cancer worry or perceived risk^36^, education attainment^37^, household income^3^, etc. Of note, a recent study reported that being aware of one’s own breast density and having a good level of breast density knowledge were positively associated with screening intentions^38^. Given that current screening uptake under the KNCSP is less than optimal, strategies to improve screening rates based on these findings need to be explored.

In the current study, we also estimated NNS as a surrogate measure for economic effects. Overall, the NNS values in our study seem to outweigh those from Western countries. In particular, the USPSTF reported NNS values of 1,904, 1,339, and 377 for women aged 40−49, 50−59, and 60−69 years, repsectively^39^. The differences in these numbers might stem from different incidence rates, screening attendance rates, and the size of the effects of mammographic screening in reducing breast cancer mortality. In particular, in younger women who had low overall mortality among both non-screened and screened women, the highest NNS value at 3,633 was estimated, indicating a substantial economic burden on the KNCSP.

The present study has several limitations. First, this study was an observational study. Therefore, we were unable to control for biases, such as self-selection bias and length bias. Accordingly, the participants in the KNCSP might over-represent healthy or health-conscious individuals, leading to an overestimation of the effectiveness of breast cancer screening. We methodologically accounted for this by estimating the magnitude of potential self-selection bias via calculation of risk reductions for all-cause deaths except from breast cancer, although this was not fully able to capture the pure screening effect. Still, our estimates of screening effects were based on high-quality real-world data, providing real-world evidence of the organized screening effect for breast cancer in Korea. Second, the design of this study may impose several inherent methodological biases, such as misclassification of exposure. As we utilized a never/ever approach, women who underwent screening once and never came back were still counted as screened women. However, whether a patient is exposed or never exposed to screening is one of the important facilitators determining her future screening behaviors^40,41^, and a number of prior studies have applied this approach^20,24,35^. Moreover, Korean women, in general, can undergo opportunistic screening at private clinics, which might result in contamination of the non-screened women in our analysis. If we consider those cases, the preventive effects of breast cancer screening would have been greater. Furthermore, breast cancer treatments, such as chemoprevention and/or adjuvant chemotherapy, have improved. Although we cannot differentiate the effects from improved treatment and screening on breast cancer mortality reduction, this should not have biased the estimates in the current study, because all Korean residents are insured by the National Health Insurance System (NHIS) and women throughout the country receive similar treatment. Fourth, we could not adjust for confounders, except for age and socioeconomic status.

The current study has several strengths. First, we conducted a nationwide cohort study with long-term follow-up to evaluate the effectiveness of an organized breast cancer screening program in Asia. Our results provide real-world evidence of organized breast cancer screening and are more generalizable to other Asian countries where women tend to have higher breast density than that in Western countries. Secondly, we utilized data on invitations and attendance in the KNCSP at the individual level, as well as on the outcomes of the screening tests. Cancer registry and mortality data were linked to the KNCSP database, and the information used in the current analysis was over 95% and 99% complete, respectively^7^.

In conclusion, the KNCSP for breast cancer with biennial mammographic screening has significantly reduced mortality from breast cancer among Korean women aged 40−69 years. The highest impact of mammographic screening was observed among women aged 45−59 years, which further implicates significant cost-effectiveness and life-years gain from the KNCSP. Our results advocate the effectiveness of an organized breast cancer screening in Asian countries, although a benefits-harms comparison and economic evaluation might be further required to estimate the validity of its implementation.

## Methods

### Study population and analytic database

In Korea, women aged 40 years and older are able to undergo two-view mammographic screening, combining craniocaudal and mediolateral oblique views, every 2 years according to the NHIS^3^. One of three mammography systems, screen-film mammography, computed radiography, and full-field digital mammography, is provided depending on the screening unit. In the current study, we selected women invited to participate in the KNCSP between 2002 and 2003 and aged 40−79 years in consideration of lower breast cancer incidence and screening participation among women over 80 years^7^. From the KNCSP database, we obtained information on each participant’s sociodemographic characteristics (age and NHIS type), screening results, and screening dates from 2002 to 2014^42^. Written informed consent was obtained from participants in the KNCSP for the collection of screening results; the requirement for informed consent for the current study was waived owing to the use of de-identified data. This study was approved by the Institutional Review Board of the National Cancer Center, Korea (IRB No.: NCCNCS08129).

The baseline cohort comprised 8,485,675 women. Among them, 1,912 women with incomplete identification numbers were excluded, as were 183,081 women with a previous diagnosis of cancer, as identified in the Korea Central Cancer Registry (KCCR), which contains information on over 95% of all newly diagnosed malignancies in Korea. Therefore, a total of 8,300,682 Korean women were included as a cancer-free cohort (Fig. 1).

### Outcome

The primary outcome of the current study was breast cancer mortality. We also examined all-cause mortality other than breast cancer to adjust for methodological bias, such as misclassification bias and competing risks. Using unique 13-digit resident registration numbers, we linked our baseline cohort from the KNCSP database (2002−2014), the KCCR for information on the date of primary breast cancer diagnosis (ICD-10: C50.0-C50.9, D05.0-D05.9) (2002−2014), and death certificates for information on the dates and causes of all mortalities (2002−2015). Regardless of women’s actual screening participation, all information from the cancer registry and death certificates were linked to the study cohort. With permission from the Ministry of Health and Welfare, the investigators used data de-identified by the NHIS.

### Exposure to screening: a modified ever/never approach

Exposure to breast cancer screening was defined using a never/ever approach by which women were considered ever screened after their first screening attendance. Women in this study cohort were defined as screened or non-screened based on the date of their first attendance to breast cancer screening^24^. Women who never underwent breast cancer screening during the follow-up period (*n* = 2,047,569) were assigned to a non-screened cohort, for which women-years were derived from the postal date (January 1, 2002; January 1, 2003) of the first screening invitation to the date of death or the end of follow up (December 31, 2015), whichever came first. Women who underwent breast cancer screening at the initial invitation were assigned to a screened cohort (*n* = 1,099,417), for which women-years were assessed as the first date of screening attendance to the date of death or the end of follow up.

Due to low screening uptake between 2002 and 2003, there was a considerable number of remaining women (*n* = 5,153,696) who did not undergo screening upon receiving their initial invitation but attended another round of screening during the follow-up period. Therefore, we accounted for the change in their exposure status from the non-screened to screened cohort as follows: the women contributed their women-years in the non-screened cohort from the date of initial invitation (January 1, 2002; January 1, 2003) to the date of their first screening attendance and in the screened cohort from the date of screening to the date of death or end of follow-up. The number of women was counted twice in both non-screened and screened cohorts, but women-years were allocated separately depending on their exposure status (i.e., the date of first screening attendance). Furthermore, when women entered into the screened cohort, the age at cohort enrollment was also updated to the age at first screening attendance accordingly. However, if women became aged over 80 years when they entered into the screened cohort, they were excluded (*n* = 106,219). Furthermore, women who were diagnosed with breast cancer before their first screening attendance (*n* = 11,598) were not changeable to the screened cohort and remained in the non-screened cohort. Women who were diagnosed with breast cancer within 90 days (*n* = 9,693) from their first screening attendance also remained in the non-screened cohort to eliminate the potential for symptom-detected breast cases. Finally, 7,201,265 women and 6,125,603 women were assigned to the non-screened and screened cohorts, respectively (Fig. 1).

### Statistical analysis

Descriptive statistics reflected the sociodemographic characteristics of the population. We used Poisson regression to estimate IRRs of invasive breast cancer, as well as IRRs of both in situ and invasive breast cancer between screened and non-screened women. MRRs of breast cancer and all-cause deaths excluding breast cancer were estimated as primary outcomes. Poisson analyses estimated IRRs and MRRs within 95% confidence intervals after the adjustment for sociodemographic factors, including age at enrollment of the cohort and National Health Insurance (NHI) status.

To exclude the possibility for self-selection bias in the cohort design, we applied the following mathematical formula^35^: net benefit = (MRR^b^ − MRR^a^)/MRR^b^ × 100, where MRR^b^ reflects the MRR for total mortality except from breast cancer deaths and MRR^a^ reflects the MRR for breast cancer mortality.

Cumulative mortality rates and Nelson−Aalen estimates of cumulative mortality were also calculated. For Nelson−Aalen estimates, we first divided “the number of breast cancer deaths in each year since cohort enrollment” by “women-years for the corresponding year”, after which the individual rates were cumulatively summed. Nelson−Aalen estimates are a more complete measure reflective of a decreased number of women-year on a yearly-basis^43^. Finally, we calculated NNS to prevent one death from breast cancer^44^. All statistical analyses were performed using SAS version 9.3 statistical software. All reported *P* values were considered statistically significant if they were less than 0.05.

### Reporting summary

Further information on research design is available in the Nature Research Reporting Summary linked to this article.

## Supplementary information

Supplementary Information

Reporting Summary

## Data Availability

The data generated and analyzed during this study are described in the following data record: 10.6084/m9.figshare.14687187^45^. The data are contained in a series of SAS data files. These files are housed on institutional file storage are the Korean National Health Insurance System (NHIS). The files are not publicly available as they contain information that could compromise research participant privacy and informed consent to share participant-level data was not obtained prior to or during data collection. Data are only available to authorized researchers who have submitted an IRB application. Details of request procedures can be found at https://nhiss.nhis.or.kr/bd/ab/bdaba021eng.do. Furthermore, according to the “Personal Information Protection Act”, datasets generated and/or analyzed during the study can only be accessible in a designated analysis center in the NHIS.

## Code availability

The codes generated during this study are available upon reasonable request. Analyses were performed using SAS (SAS Institute Inc., Cary, NC, USA) version 9.3.
