# Supplementary material for: Effectiveness of the Korean National Cancer Screening Program in reducing breast cancer mortality
Source: NPJ Breast Cancer. 2021 Jun 28;7:83. doi: 10.1038/s41523-021-00295-9 (PMC8238931; doi:10.1038/s41523-021-00295-9)
Supplement: Supplementary file 1 — Supplementary Information [file 41523_2021_295_MOESM1_ESM.pdf]

Supplementary Materials

Supplementary Table 1. Number of screening attendances among women in the screened cohort

Supplementary Table 2. Year at first screening attendance among women in the changeable group

Supplementary Table 3. Characteristics of newly diagnosed breast cancers between screened and non-screened women in the KNCSP, 2002-2014

Supplementary Table 4. Cumulative number and cumulative incidence rates of invasive and in situ breast cancer in the screened and non-screened women in the KNCSP, 2002-2014

Supplementary Table 5. Cumulative breast cancer mortality rates and Nelson-Aalen estimates of cumulative breast cancer mortality rates of the screened and non-screened women in the KNCSP, 2002-2015

Supplementary Table 6. Number needed to screen (NNS) to save one life in the KNCSP for breast cancer

Supplementary Table 1. Number of screening attendances among women in the screened cohort[a]

No. of screening attendance	N	(%)
1	1,456,178	23.77
2	1,287,002	21.01
3	1,324,383	21.62
4	1,195,255	19.51
5	675,315	11.02
6+	187,470	3.06

[a] Total number of women in the screened cohort (N=6,125,603) (See Table 1).

Supplementary Table 2. Year at first screening attendance among women in the changeable group[a]

Year at screening attendance[b]	N	(%)
2004	430,088	(8.56)
2005	619,238	(12.32)
2006	837,988	(16.67)
2007	721,863	(14.36)
2008	653,181	(13.00)
2009	565,586	(11.25)
2010	409,018	(8.14)
2011	308,528	(6.14)
2012	218,728	(4.35)
2013	148,133	(2.95)
2014	113,835	(2.26)

[a] Total number of screened women (N=5,026,186) in the changeable group (See Figure 1).

[b] Women with even year-of-birth were invited to the biennial mammographic screening at even-year; women with odd year-of-birth were invited at odd-year.

Supplementary Table 3. Characteristics of newly diagnosed breast cancers between screened and non-screened women in the KNCSP, 2002-2014

	Screened cohort		Non-screened cohort		*P* value
	N	(%)	N	(%)	
Total incident cases	50,054	(100.00)	49,050	(100.00)	
Age at diagnosis of breast cancer (Years; SD)	57.60 (8.37)		55.40 (9.60)		<.0001
5-Years age groups at diagnosis of breast cancer					
40-44	1,133	(2.26)	4,758	(9.70)	<.0001
45-49	7,623	(15.23)	12,357	(25.19)	
50-54	14,435	(28.84)	11,577	(23.60)	
55-59	10,024	(20.03)	6,923	(14.11)	
60-64	7,073	(14.13)	5,062	(10.32)	
65-69	4,664	(9.32)	3,445	(7.02)	
70-74	3,211	(6.42)	2,534	(5.17)	
75-79	1,891	(3.78)	2,394	(4.88)	
National Health Insurance					
Upper 50%	31,211	(62.35)	32,506	(66.27)	<.0001
Lower 50%	16,759	(33.48)	14,885	(30.35)	
Medical Aid Program	2,084	(4.16)	1,659	(3.38)	
Incidence-based mortality[a]					
Breast cancer	2,694	(5.38)	6,626	(13.51)	<.0001
Others	780	(1.56)	1,765	(3.60)	
Alive	46,580	(93.06)	40,659	(82.89)	
Cancer histologic type					
In situ breast cancer[b]	6,723	(13.43)	4,469	(9.11)	<.0001
Invasive breast cancer	43,331	(86.57)	44,581	(90.89)	
SEER[c]					
Localized + in situ breast cancer[b]	30,957	(64.74)	20,166	(54.50)	<.0001
Regional	13,033	(27.26)	10,995	(29.72)	
Distant	1,325	(2.77)	2,213	(5.98)	
Unknown	2,502	(5.23)	3,627	(9.80)	
SEER[c] for invasive breast cancer					
Localized	24,234	(59.0)	15.697	(48.3)	<.0001
Regional	13,033	(31.7)	10,995	(33.8)	
Distant	1,325	(3.2)	2,213	(6.8)	
Unknown	2,502	(6.1)	3,627	(11.1)	

Abbreviations: SD, standard deviation.

[a] During 2002-2015

[b] Lobular carcinoma included

[c] 2,237 (screened cohort) and 12,049 (non-screened cohort) missing values detected in the screened and non-screened cohorts during 2002 and 2004

Supplementary Table 4. Cumulative number and cumulative incidence rates of invasive and in situ breast cancer in the screened and non-screened women in the KNCSP, 2002-2014

Year since inclusion in the cohort	Screened cohort (N = 6,125,603)				Non-screened cohort (N = 7,201,265)			
	Cumulative No. of invasive breast cancers	(Rates[b])	Cumulative No. of in situ breast cancers[a]	(Rates[b])	Cumulative No. of invasive breast cancers	(Rates[b])	Cumulative No. of in situ breast cancers[a]	(Rates[b])
1	4708	(76.86)	680	(11.10)	4887	(67.86)	359	(4.99)
2	10090	(164.72)	1383	(22.58)	9623	(133.63)	790	(10.97)
3	15610	(254.83)	2247	(36.68)	15094	(209.60)	1299	(18.04)
4	20835	(340.13)	3052	(49.82)	19748	(274.23)	1749	(24.29)
5	25811	(421.36)	3846	(62.79)	24587	(341.43)	2279	(31.65)
6	30282	(494.35)	4550	(74.28)	28510	(395.90)	2716	(37.72)
7	34088	(556.48)	5174	(84.47)	32333	(448.99)	3186	(44.24)
8	37335	(609.49)	5674	(92.63)	35351	(490.90)	3489	(48.45)
9	39789	(649.55)	6110	(99.75)	38246	(531.10)	3805	(52.84)
10	41428	(676.31)	6398	(104.45)	40324	(559.96)	4000	(55.55)
11	42592	(695.31)	6605	(107.83)	42309	(587.52)	4231	(58.75)
12	43189	(705.06)	6699	(109.36)	43833	(608.68)	4398	(61.07)
13	43331	(707.38)	6723	(109.75)	44581	(619.07)	4469	(62.06)

[a] Lobular carcinoma included.

[b] Per 100,000 women

Supplementary Table 5. Cumulative breast cancer mortality rates and Nelson-Aalen estimates of cumulative breast cancer mortality rates of the screened and non-screened women in the KNCSP, 2002-2015

Year since inclusion in the cohort	Cumulative breast cancer mortality rates[a] per 100,000 women (95% CI)				Nelson-Aalen estimates of cumulative breast cancer mortality rates[b] per 100,000 women-years (95% CI)			
	Screened cohort		Non-screened cohort		Screened cohort		Non-screened cohort	
1	0.56	(0.37-0.74)	1.18	(0.93-1.43)	0.56	(0.37-0.74)	1.18	(0.93-1.43)
2	2.40	(1.86-2.94)	3.99	(3.52-4.45)	2.43	(1.90-2.96)	4.02	(3.38-4.66)
3	5.45	(4.63-6.27)	9.46	(8.75-10.17)	5.61	(4.62-6.60)	9.89	(8.67-11.12)
4	9.94	(8.84-11.05)	16.12	(15.19-17.05)	10.46	(8.90-12.02)	17.97	(16.03-19.92)
5	15.35	(13.97-16.72)	23.51	(22.39-24.63)	16.61	(14.39-18.84)	28.18	(25.37-31.00)
6	21.32	(19.70-22.94)	32.05	(30.74-33.36)	23.96	(20.98-26.93)	42.31	(38.38-46.24)
7	27.47	(25.64-29.31)	41.10	(39.62-42.58)	32.38	(28.56-36.21)	59.91	(54.63-65.19)
8	33.19	(31.17-35.21)	50.87	(49.22-52.51)	41.47	(36.70-46.25)	82.89	(75.91-89.86)
9	38.04	(35.88-40.20)	60.67	(58.87-62.47)	50.82	(44.98-56.67)	109.82	(100.85-118.78)
10	42.04	(39.76-44.31)	70.50	(68.56-72.44)	61.01	(53.89-68.13)	142.15	(130.80-153.49)
11	44.80	(42.45-47.14)	79.32	(77.26-81.38)	71.21	(62.55-79.87)	174.23	(160.39-188.07)
12	47.23	(44.82-49.64)	88.28	(86.11-90.45)	83.79	(73.11-94.47)	211.49	(194.77-228.21)
13	48.49	(46.04-50.93)	97.12	(94.85-99.40)	96.27	(82.81-109.73)	250.65	(230.89-270.41)
14	48.88	(46.43-51.33)	100.97	(98.65-103.29)	116.73	(95.08-138.38)	290.81	(266.32-315.29)

[a] Cumulative mortality rates: the total number of deaths from breast cancer at each year since randomization divided by the total number of women in each group.

[b] Nelson-Aalen estimates of cumulative mortality rate: the number of breast cancer deaths in each year since randomization divided by the number of women-years observed during that year and by summing these individual rates.

Supplementary Table 6. Number needed to screen (NNS) to save one life in the KNCSP for breast cancer

	Mortality rates from breast cancer (per 100,000 women)		Absolute risk reduction	NNS
	Non-screened cohort	Screened cohort		
Total	100.97	48.88	52.09	1,920
5-Year age group				
40-44	97.38	69.85	27.52	3,633
45-49	111.34	52.21	59.13	1,691
50-54	106.40	44.77	61.63	1,623
55-59	104.59	45.33	59.25	1,688
60-64	91.12	45.34	45.78	2,184
65-69	98.72	42.40	56.32	1,776
70-74	93.73	48.18	45.56	2,195
75-79	107.61	54.21	53.39	1,873
National Health Insurance				
Upper 50%	90.47	42.38	48.08	2,080
Lower 50%	112.15	53.86	58.29	1,715
Medical Aid Program	164.67	94.08	70.60	1,416

Abbreviation: NNS, number needed to screen.
