# Supplementary material for: Effectiveness of the Korean National Cancer Screening Program in reducing breast cancer mortality
Source: NPJ Breast Cancer. 2021 Jun 28;7:83. doi: 10.1038/s41523-021-00295-9 (PMC8238931; doi:10.1038/s41523-021-00295-9)
Supplement: Supplementary file 2 — Reporting Summary [file 41523_2021_295_MOESM2_ESM.pdf]

nature research

Corresponding author(s): Kui Son Choi

Last updated by author(s): May 6, 2021

Reporting Summary

Nature Research wishes to improve the reproducibility of the work that we publish. This form provides structure for consistency and transparency in reporting. For further information on Nature Research policies, see our Editorial Policies and the Editorial Policy Checklist.

Statistics

For all statistical analyses, confirm that the following items are present in the figure legend, table legend, main text, or Methods section.

n/a	Confirmed	
☐	☒	The exact sample size (*n*) for each experimental group/condition, given as a discrete number and unit of measurement
☒	☐	A statement on whether measurements were taken from distinct samples or whether the same sample was measured repeatedly
☐	☒	The statistical test(s) used AND whether they are one- or two-sided<br>*Only common tests should be described solely by name; describe more complex techniques in the Methods section.*
☐	☒	A description of all covariates tested
☒	☐	A description of any assumptions or corrections, such as tests of normality and adjustment for multiple comparisons
☐	☒	A full description of the statistical parameters including central tendency (e.g. means) or other basic estimates (e.g. regression coefficient) AND variation (e.g. standard deviation) or associated estimates of uncertainty (e.g. confidence intervals)
☐	☒	For null hypothesis testing, the test statistic (e.g. *F*, *t*, *r*) with confidence intervals, effect sizes, degrees of freedom and *P* value noted<br>*Give P values as exact values whenever suitable.*
☒	☐	For Bayesian analysis, information on the choice of priors and Markov chain Monte Carlo settings
☒	☐	For hierarchical and complex designs, identification of the appropriate level for tests and full reporting of outcomes
☐	☒	Estimates of effect sizes (e.g. Cohen's *d*, Pearson's *r*), indicating how they were calculated

Our web collection on statistics for biologists contains articles on many of the points above.

Software and code

Policy information about availability of computer code

Data collection	Breast cancer screening database from the Korean National Cancer Screening Program (KNCSP), cancer registry data from the Korea Central Cancer Registry (KCCR) and death certificates were provided as de-identified text data with a random matching ID by the National Health Insurance System (NHIS). Data merging and analyses were performed using SAS (SAS Institute Inc., Cary, NC, USA) version 9.3.
Data analysis	A description of the software and code has been included in the Methods. We used Poisson regression to estimate incidence rate ratios of invasive and in situ breast cancer between screened and non-screened women. Mortality rate ratio (MRR) of breast cancer and all-cause deaths excluding breast cancer between screened and non-screened women were also estimated using Poisson regression with adjustment for sociodemographic factors. Analyses were performed using SAS (SAS Institute Inc., Cary, NC, USA) version 9.3. To exclude the possibility for self-selection bias in the cohort design, we applied the following mathematical formula: net benefit = [(MRR for total mortality except from breast cancer deaths)-(MRR for breast cancer mortality)]/ (MRR for total mortality except from breast cancer deaths) X100.

For manuscripts utilizing custom algorithms or software that are central to the research but not yet described in published literature, software must be made available to editors and reviewers. We strongly encourage code deposition in a community repository (e.g. GitHub). See the Nature Research guidelines for submitting code & software for further information.

Data

Policy information about availability of data

All manuscripts must include a data availability statement. This statement should provide the following information, where applicable:

- Accession codes, unique identifiers, or web links for publicly available datasets
- A list of figures that have associated raw data
- A description of any restrictions on data availability

The data sets generated and/or analyzed during the current study are not publicly available.

Field-specific reporting

Please select the one below that is the best fit for your research. If you are not sure, read the appropriate sections before making your selection.

☐ Life sciences ☒ Behavioural & social sciences ☐ Ecological, evolutionary & environmental sciences

For a reference copy of the document with all sections, see nature.com/documents/nr-reporting-summary-flat.pdf

Behavioural & social sciences study design

All studies must disclose on these points even when the disclosure is negative.

Study description	Prospective cohort study
Research sample	Data included Korean women aged 40-79 years, who were invited to the Korean National Cancer Screening Program (KNCSP) for breast cancer between 2002 and 2003 (N=8,300,682).
Sampling strategy	This is a nationwide prospective study that includes all Korean women invited to breast cancer screening services through the Korean National Cancer Screening Program (KNCSP). Although our study population was not selected by randomization procedure, the evidence obtained from our cohort design ought to reflect the real-world effectiveness of the screening program for breast cancer mortality reduction.
Data collection	Korean women aged 40 years and older are eligible to the breast cancer screening through the Korean National Cancer Screening Program (KNCSP) and automatically enrolled in the screening database. For whom underwent breast cancer screening, screening records and results were accumulated. Regardless of women's screening attendance status, detailed information of incident breast cancers and deaths were identified through linkage to the Korean Central Cancer Registry (KCCR) and death certificates, respectively.
Timing	Our baseline cohort was comprised of women who were invited to the Korean National Cancer Screening Program (KNCSP) for breast cancer between 2002 and 2003. They were followed through 2014 for incident breast cancers, and through 2015 for deaths.
Data exclusions	The baseline cohort comprised 8,485,675 women. Among them, 1,912 women with incomplete identification numbers were excluded, as were 183,081 women with a previous diagnosis of cancer, as identified in the Korea Central Cancer Registry (KCCR), which contains information on over 95% of all newly diagnosed malignancies in Korea. Therefore, a total of 8,300,682 Korean women were included as a cancer-free cohort.
Non-participation	All women in the study cohort were followed up to the date of death or the end of follow up, whichever came first.
Randomization	Exposure to breast cancer screening was basically defined using a never/ever approach by which women were considered ever screened after their first screening attendance. Women in this study cohort were defined as screened or non-screened based on the date of their first attendance to breast cancer screening. To account for potential selection bias, we applied a modification to the never/ever approach by using a changeable group, and further performed mathematical adjustment using breast cancer-specific and all-cause except breast cancer mortality rates.

Reporting for specific materials, systems and methods

We require information from authors about some types of materials, experimental systems and methods used in many studies. Here, indicate whether each material, system or method listed is relevant to your study. If you are not sure if a list item applies to your research, read the appropriate section before selecting a response.

Materials & experimental systems

n/a	Involved in the study	
☒	☐	Antibodies
☒	☐	Eukaryotic cell lines
☒	☐	Palaeontology and archaeology
☒	☐	Animals and other organisms
☐	☒	Human research participants
☒	☐	Clinical data
☒	☐	Dual use research of concern

Methods

n/a	Involved in the study	
☒	☐	ChIP-seq
☒	☐	Flow cytometry
☒	☐	MRI-based neuroimaging

Human research participants

Policy information about studies involving human research participants

Population characteristics	"See above."

Recruitment	Korean women aged 40 years and older are eligible to the breast cancer screening services through the Korean National Cancer Screening Program (KNCSP) and automatically enrolled in the screening database. For whom underwent breast cancer screening, screening records and results are accumulated. Regardless of screening attendance, the eligible women in the screening database are followed through 2015, by linkage to cancer registry data and death certificates.
Ethics oversight	This study was approved by the Institutional Review Board of the National Cancer Center, Korea (IRB No.: NCCNCS08129). With permission from the Ministry of Health and Welfare, the investigators received de-identified screening database, cancer registry and death certificates for the study period through the National Health Insurance System.

Note that full information on the approval of the study protocol must also be provided in the manuscript.
